# Transcriptomic reprogramming and epigenetic regulation underlying pollination-dependent and auxin-induced fruit set in tomato

**DOI:** 10.3389/fpls.2025.1495494

**Published:** 2025-02-11

**Authors:** Xiaohan Li, Bing He, Anis Djari, Pierre Frasse, Elie Maza, Farid Regad, Julien Pirrello, Guojian Hu, Mondher Bouzayen

**Affiliations:** ^1^ College of Agriculture and Biotechnology, Zhejiang University, Hangzhou, China; ^2^ Zhejiang Provincial Key Laboratory of Horticultural Plant Integrative Biology, Zhejiang University, Hangzhou, China; ^3^ The State Agriculture Ministry Laboratory of Horticultural Plant Growth, Development and Quality Improvement, Zhejiang University, Hangzhou, China; ^4^ College of Horticulture, China Agricultural University, Beijing, China; ^5^ Laboratoire de Recherche en Sciences Végétales—Génomique et Biotechnologie des Fruits—UMR5546, Université de Toulouse, Centre national de la recherche scientifique (CNRS), Université de Toulouse 3 - Paul Sabatier (UPS), Toulouse-Institut National Polytechnique (INP), Toulouse, France

**Keywords:** fruit set, auxin, pollination, transcriptomic reprogramming, epigenetic regulation, tomato

## Abstract

The transition from flower to fruit, naturally triggered by flower pollination and known as fruit set, is instrumental for plant reproduction, seed formation, and crop yield. Notably, this developmental process can also proceed in the absence of flower fertilization, although it remains unclear whether pollination-dependent and pollination-independent fruit sets undergo similar transcriptomic reprogramming. Genome-wide transcriptomic profiling of the flower-to-fruit transition, either pollination-induced or triggered by auxin treatment, shows that both types of triggers modulate the expression of a common large set of genes primarily expressed in maternal tissues. These include genes related to auxin, gibberellin, brassinosteroid, and ethylene signaling. Furthermore, analysis of changes in histone marking during this transition phase indicated that gene reprogramming underlying both types of fruit set primarily correlated with dynamic changes in H3K9ac and H3K4me3 histone marks. Notably, *MCM1, AG, DEFA and SRF (MADS)-box* and *NAM, ATAF1/2, and CUC2 (NAC)* genes were extensively downregulated during the transition from flower to fruit, suggesting their negative roles in fruit initiation. In contrast, *Teosinte branched1/Cincinnata/proliferating cell factor (TCP), SQUAMOSA -promoter binding proteins (SBP), Sucrose nonfermenting 2 (SNF2), Growth-regulating factor (GRF), and Su (var)3-9, Enhancer-of-zeste and Trithorax (SET)* family genes were significantly upregulated in both pollinated and auxin-treated young developing fruits, suggesting their active roles in promoting fruit sets. Despite these similarities, a comparative analysis of the effects of natural pollination and auxin treatment revealed several differences, primarily related to seed development and hormone signaling. Taken together, the data support the idea that auxin serves as the central hormone orchestrating the extensive gene reprogramming associated with fruit initiation in tomato.

## Introduction

Fruit set, an essential transition from flower opening to young fruit development, is naturally triggered by ovule fertilization, which coordinately activates developmental programs, including seed development and the growth of various peripheral structures that protect the developing seeds (Pandolfini et al., 2007). During the anthesis stage, the unpollinated ovary enters a temporary growth arrest until flower fertilization occurs, triggering a developmental switch that forms the arrested ovary into a rapidly growing fruit. Underpinning this developmental transition, cell division is rapidly initiated in the placenta and pericarp tissues following successful ovule fertilization. While it is generally accepted that fertilized ovules, now developing into young seeds, are the primary structures releasing signals that trigger cell division and fruit growth in various plant species, the molecular nature of these signal(s) and their diffusion into the surrounding tissues remain poorly understood.

Phytohormones play a pivotal role in transitioning an arrested ovary into a growing fruit. Several lines of evidence single-out auxin and gibberellins (GA) as prominent positive regulators of fruit set, as the application of these hormone substrates to unpollinated ovaries stimulates parthenocarpic fruit formation in various plant species (Gustafson, 1936; Bünger-Kibler and Bangerth, 1982; Pandolfini et al., 2007; Serrani et al., 2008; de Jong et al., 2009a). Direct evidence that auxin triggers fruit set has been explored across physiological, biochemical, and molecular levels, encompassing auxin biosynthesis, metabolism, polar transport, perception, signal transduction, and responses. For instance, increasing levels of auxin through the expression of the *Pseudomonas* pv. *savastanoi iaaM* gene in the ovule or the *Agrobacterium rhizogenes rolB* gene in the ovary has been shown to promote parthenocarpic fruit formation in several horticultural crops (Rotino et al., 1997, 2005; Donzella et al., 2000; Pandolfini et al., 2002; Carmi et al., 2003; Mezzetti et al., 2004). Similarly, knocking down the expression of the auxin efflux carrier *SlPIN4* gene results in parthenocarpic fruit setting (Mounet et al., 2012), and altering auxin perception through the overexpression of *TRANSPORT INHIBITOR 1* (*SlTIR1*) also induces parthenocarpy (Ren et al., 2011). More recently, impairing the expression of several components of the auxin signaling pathway, including *IAA9*, *SlARF7*, and *ARF8A/B* has been reported to result in seedless fruit setting (Wang et al., 2005; Goetz et al., 2007; de Jong et al., 2009b; Hu et al., 2023). Altogether these data clearly emphasize the primary role of auxin during the flower-to-fruit transition. GAs are another factor controlling fruit set and subsequent fruit development. High levels of GA have been reported in parthenocarpic tomato lines *pat-2* and *pat-3/pat-4* (Fos et al., 2000, 2001), and inactivation of *GA 2-oxidase* genes leads to the formation of parthenocarpic fruits in *Arabidopsis* (Rieu et al., 2008). Similarly, the alternation of the active GA form by overexpression of the citrus *CcGA20ox1* gene in tomato triggers fruit growth in the absence of pollination (Garcia-Hurtado et al., 2012). Activation of the GA signaling pathway relies on the presence of GA3, which stimulates the ubiquitin-dependent proteolytic degradation of the nuclear repressor DELLA by the 26S proteasome, thereby releasing the repression of GA response genes by DELLA (Davière and Achard, 2013). Along the same line, downregulation of the *SlDELLA* gene expression, which encodes a negative regulator of GA signaling, induces parthenocarpic fruit formation and reduced fruit size in tomato (Martí et al., 2007). Taken together, these data support the notion that active GA signaling promotes fruit set and ovary growth. Furthermore, auxin has been reported to positively regulate the expression of GA biosynthesis genes in the ovules, leading to GA accumulation, suggesting that auxin acts prior to GA in promoting fruit initiation. Accordingly, transcripts corresponding to the copalyldiphosphate synthase (*SlCPS*), *SlGA20ox1*, *SlGA20ox2*, *SlGA20ox3*, and *SlGA3ox1* genes accumulate to higher levels in 2,4-D-treated ovaries, while transcript levels of GA-inactivating enzyme *SlGA2ox2* are decreased (Dorcey et al., 2009). In addition, the gaseous hormone ethylene also appears to influence fruit set, as ethylene production decreases in pollination-induced and in pollination-independent fruit set in the auxin hypersensitive tomato mutant *iaa9-3* (Shinozaki et al., 2015). Accordingly, mutation of the ethylene perception gene *Sletr1-1* in tomato leads to elevated levels of bioactive GAs the formation of elongated parthenocarpic fruit, suggesting that ethylene plays a role in maintaining ovary growth arrest prior to pollination by suppressing GA metabolism. Altogether, this reveals the complexity of the hormonal regulatory network underlying the fruit-set process.

In addition to the central role of hormones, the flower-to-fruit transition involves the intervention of several developmental factors, most of which are transcription factors (TFs). For instance, loss-of-function of the *PI* MADS-box gene in apples produces *apetalous* flowers and seedless fruits (Yao et al., 2001). Silencing of the *SEPALLATA* (*SEP*) MADS-box gene *TM29* leads to parthenocarpic fruit formation in tomato (Ampomah-Dwamena et al., 2002). Two other MADS-box genes, *Tomato Agamous1* (*TAG1*) and *Tomato Agamous6* (*TAGL6*), were suggested to play negative roles in fruit setting, based on the observation that their transcript levels dramatically decrease during both pollination-induced and pollination-independent fruit set in wild-type and in *IAA9* tomato mutants, respectively (Wang et al., 2009). In addition, miR156 and its target SQUAMOSA promoter-binding protein-like (SPL or SBP-box) genes were found to be differentially expressed in pre- and postanthesis ovaries. Overexpression of *AtMIR156* resulted in partial seedless fruit formation, defining miR156 and SPL transcription factors as a regulatory module controlling the early stages of fruit development (Silva et al., 2014). Moreover, the transcript levels of *GROWTH REGULATING FACTOR 2* (*GRF2*), a member of the transcription activator gene family, were shown by cDNA-amplified fragment length polymorphism (AFLP) to increase in pollinated ovaries (Vriezen et al., 2007). Although our understanding of the central roles of hormones and transcription factors in fruit setting has progressed tremendously in recent decades, the similarities and differences between the molecular mechanisms of the hormonal signaling and transcriptomic reprogramming involved in pollination-dependent and pollination-independent fruit setting have largely been overlooked.

Epigenetic marking, including DNA methylation of 5′ cytosine residues and posttranslational modification of histones (Henderson and Jacobsen, 2007), appears to be the main mechanism regulating gene expression during developmental transitions underlying organ and tissue differentiation, as well as plant reproduction in living organisms (Pu and Sung, 2015; Gehring, 2019). In plants, histone modifications are a major factor in controlling transitions across various developmental stages, including the circadian clock, stress response (Berr et al., 2011; Malapeira et al., 2012), and fruit set induced by pollination (Hu et al., 2021). Extensive research has demonstrated that the biosynthesis, transport, and signaling of phytohormones are regulated by histone modifications (Rudolf et al., 2024). For instance, H3K27me3 acts as a repressive mark across large genomic regions for genes involved in auxin metabolism and transport, such as *YUCCA* (*YUC*), *CYTOCHROME P450* (*CYP*), and *TRYPTOPHAN AMINOTRANSFERASE 1/TRYPTOPHAN AMINOTRANSFERASE-RELATED* (*TAA1/TAR*) and *PIN-FORMED* (*PINs*) genes (Lafos et al., 2011; He et al., 2012). Importantly, the POLYCOMB REPRESSIVE COMPLEX2 (PRC2)-mediated FERTILIZATION INDEPENDENT (FIS-PRC2) complex has been shown to block the expression of auxin biosynthesis and gibberellin-related genes in unfertilized ovules through H3K27me3 deposition. This repression is lifted upon fertilization, leading to paternal expression of auxin biosynthesis genes, which promotes endosperm formation and seed set in *Arabidopsis* (Figueiredo et al., 2015, 2016). Furthermore, transcription factors can reshape the epigenetic state of the chromatin regions they bind to, either by facilitating the binding of other transcription factors or by directly recruiting histone modifiers (Soufi et al., 2012). For instance, *TCP5* regulates the transition from cell division to postmitotic expansion of petal primordia in *Arabidopsis* (Huang and Irish, 2015). The RABBIT EARS (RBE) transcriptional repressor maintains the downregulation of its direct target, *TCP5*, by recruiting the TOPLESS (TPL)-HDA19 corepressor complex to inhibit *TCP5* transcription (Huang and Irish, 2024). The reduced transcription of *TCP5* is associated with a decrease in H3K9ac and an increase in H3K27me3 histone marks. It is noteworthy that silencing the corepressor *SlTPL1* induces facultative parthenocarpic fruit formation in tomato (He et al., 2021), a phenotype similar to that observed in the antisense line of its partner, *IAA9*. Altogether, these studies reveal the interplay among hormones, TFs, and epigenetic mechanisms, underscoring their critical role in driving developmental transitions in plants.

Using combined genome-wide transcriptomic profiling and Chromatin immunoprecipitation followed by sequencing (ChIP-seq) strategies, we previously showed that histone marking, rather than DNA methylation, is strongly correlated with the transcriptomic reprogramming underpinning fruit set in tomato, with H3K9ac and H3K4me3 permissive marks being the primary players in this control mechanism (Hu et al., 2021). Considering that auxin can trigger fruit set independently of pollination, we sought to comparatively investigate whether histone marking plays a role in both auxin-mediated natural pollination-induced fruit setting. The outcome of the study supports the notion that both auxin-induced and pollination-triggered fruit sets rely on the expression of a common large set of genes, primarily expressed in maternal tissues and that the two types of fruit set correlate primarily with the dynamic changes of H3K9ac and H3K4me3 histone marks.

## Materials and methods

### Plant materials and sampling

All plants used in this study were *Solanum lycopersicum* L. cv MicroTom. The seeds were directly sown in soil and grown under standard culture chamber conditions as follows: 14-h-day/10-h-night cycle, 25/20°C day/night temperature, 80% relative humidity, 250 mol m^−2^ s^−1^ light intensity.

Ovary samples at 0 days postpollination (0 DPA) correspond to the anthesis stage when the stamens were loosely enclosed by petals. Fruits at 4 days postpollination (4 DPA) correspond to 4 days postanthesis. For the 4 days after auxin treatment (4 IAA) fruit samples, the flowers were first emasculated 1 day before anthesis (to avoid accidental self-pollination). From anthesis and for the next 4 days, the ovaries were treated daily with 10 μL of 500 μM indole-3-acetic acid (IAA; Sigma Aldrich, US). This treatment is considered appropriate because both pollination- and IAA-treated fruits ultimately reach similar fruit sizes at 4 and 9 days after anthesis (Hu et al., 2021). Each biological replicate corresponds to a pool of at least 50 ovaries (fruits) from 25 plants.

### RNA sample preparation and sequencing

Total RNA was extracted from ~ 200 mg of tissue for each sample using the TRIzol RNA Isolation Kit (Thermo Fisher Scientific, US). After DNA removal (DNA-free™ DNA Removal Kit, Ambion, US), RNA was purified, and its quality was checked using an Agilent 2100 analyzer. Only samples with an RIN > 8.6 were used for Illumina sequencing. Eight biological replicates were performed for each sampling stage. Paired-end RNA sequencing (2 nt × 125 nt) was performed using a Truseq Illumina SBS Kit V4 and a Hiseq2500 platform.

### RNA-seq data processing

Raw paired-end RNA-seq sequences in FASTQ format were analyzed. Low-quality reads were removed using the FASTQ quality filter from the FASTX Toolkit version 0.0.13 (http://hannonlab.cshl.edu/fastx_toolkit/). Trimmed reads were then mapped to the *S. lycopersicum* reference genome and gene annotation (ITAG4.1 Tomato_Genome_Consortium, 2012, https://solgenomics.net/) using TopHat-2.0.14 (Trapnell et al., 2009), which calls Bowtie 2.1.0 (Langmead and Salzberg, 2012). To perform differential gene analysis, HTSeq (Anders et al., 2015) was used to calculate raw counts. Raw counts of 34,727 tomato genes were normalized, and mean counts per kilobase of the transcript were used as gene expression. Differentially expressed genes between 4 DPA and 0 DPA tissues or 4 IAA and 0 DPA tissues were identified using DEseq2 (Love et al., 2014). Raw *p*-values were adjusted as “*padj*” by multiple tests using the methods of Benjamini and Hochberg (1995). Genes with |log2Fold| > 1 and *padj* < 0.01 were defined as significantly differentially expressed genes.

### Chromatin immunoprecipitation and sequencing

The experiment was performed as previously described (Hu et al., 2021). Tissues at 0 DPA, 4 DPA, and 4 IAA were cross-linked by vacuum infiltration (760 mmHg) for 15 min in 1% formaldehyde fresh 1 × PBS solution (with 0.015% Triton X-100). To ensure efficient crosslinking, 4 DPA or 4 IAA fruits were cut in half prior to crosslinking. Crosslinking was stopped by adding glycine (0.125 M final concentration) and incubating under vacuum infiltration for an additional 5 min. After washing twice with cold 1 × PBS solution, samples were thoroughly dried between paper towels, snap-frozen in liquid nitrogen, and stored at − 80°C. ChIP assays were performed as described previously (Gendrel et al., 2005) with minor modifications. Briefly, ~1 g of crosslinked tissue was ground to a fine powder in liquid nitrogen. Shearing of the chromatin was achieved through Diagenode Bioruptor sonication (5 runs of 10 cycles: 30 s “ON” and 30 s “OFF”). The size of the sonicated chromatin was checked to ensure that it was within the range of 100–500 bp. Subsequently, 10 μL of sonicated supernatant was kept aside as input. For each sample (120 μL supernatant), a dilution buffer was added to bring the final volume to 1.5 mL. Depending on the histone mark, either 5 μL of H3K9ac rabbit polyclonal antibody (Millipore, US; Cat. No. 07-352; Lot No. 2586454), 5 µL of H3K4me3 rabbit polyclonal antibody (Millipore; Cat. No. 07-473; Lot No. 2430389), or 8 μL of H3K27me3 rabbit polyclonal antibody (Millipore; Cat. No. 07-449; Lot No. 2475696) were added prior to incubation overnight (4°C at 10 rpm). For the control experiment without histone mark antibodies, 5 μL of nonimmunized rabbit IgG antibody (Millipore; Cat. No. 12-370; Lot No. 2426484) was added. For the empty control (Mock), no antibody was added. Afterward, 50 μL of protein A/G agarose beads (Pierce™ Protein A/G UltraLink™ Resin; Thermo Scientific; Cat. No. 53133) was added, and the samples were incubated for 3 h at 4°C. Beads were then sequentially washed with low salt wash buffer, high salt wash buffer, LiCl wash buffer, and finally with TE buffer. Elution was performed as previously described (Gendrel et al., 2005). Eluates of immunoprecipitated samples (IP) and input samples not subjected to immunoprecipitation were first reverse-crosslinked at 65°C overnight and then treated with 20 mg proteinase K (Invitrogen, US) for 3 h, followed by phenol/chloroform extraction, and ethanol precipitation in the presence of NaCl (3 M: pH 5.2) and glycogen. The precipitated DNA was resuspended in 10 μL of nuclease-free water and quantified by Qubit Fluorometer (Qubit dsDNA HS Assay Kit Cat. No. Q32851, Molecular Probes, US). For each sample, 10 ng of immunoprecipitated DNA was used for library construction and sequencing.

### ChIP-seq data processing

ChIP-seq read alignment was performed using Bowtie2 with default parameters, and only uniquely aligned reads were retained. Enriched regions in the nonredundant mapped reads were identified by MACS2 v1.4.2 (Zhang et al., 2008) (effective genome size = 770 Mb, *p*-value cutoff = 1.00*e*−05). Heatmap representations of signal intensity (computeMatrix scale-regions followed by plotHeatmap) were generated using the deepTools suite (Ramírez et al., 2014). The BEDtools package (Quinlan and Hall, 2010) was used to detect the tomato genes (ITAG4.1) overlapping with the detected peaks. A matrix of genes intersecting with peaks for each sample was created for downstream analyses using R software (www.r-project.org/). Differentially associated peaks were normalized and identified using the “MAnorm” method (Shao et al., 2012). For this method, the normalized *M*-value (*M* = log2 [read density in 4 IAA samples/read density in 0 DPA sample]) represents log2-transformed fold changes in enrichment intensities at each peak region. Only regions with *p*-value < 0.01 were defined as differentially associated regions (DA) (**Supplementary Table S4**).

### Gene ontology analysis

Gene ontology (GO) analysis of differentially expressed genes (DEGs) (*padj* < 0.05) was performed using PANTHER GO. Significantly enriched GO categories were selected with a false discovery rate (FDR) < 0.05.

### Quantitative RT-PCR

Total RNA extraction, genomic DNA removal, cDNA generation, and qRT-PCR were performed as previously described (Hao et al., 2015). The comparative threshold cycle method (ΔΔCt) was used for quantitative PCR (LightCycler ^®^ 480 II system, Roche, US). Tomato Actin (*Solyc11g005330*) was used as an internal reference. Primers for qRT-PCR analysis are listed in **Supplementary Table S5**. Three independent biological replicates were performed.

### Accession numbers

The datasets supporting the conclusions of this article are available (study PRJEB19602) from the European Nucleotide Archive (http://www.ebi.ac.uk/ena/data/view/PRJEB19602) with the following accession numbers: ERS1572545, ERS1572546, ERS1572547, ERS1572553, ERS1572554, ERS1572555, ERS1572556, ERS1572557, and ERS1572558 for RNA-seq analysis; ERS1572559, ERS1572560, ERS1572561, ERS1572562, ERS1572563, ERS1572564, ERS1572565, ERS1572566, ERS1572567, ERS1572568, ERS1572569, and ERS1572570 for ChIP-seq analysis.

## Results

### Global transcriptomic changes associated with auxin-induced and pollination-dependent fruit set

Fruit set is naturally triggered upon flower pollination and fertilization, and this genetically programmed process involves the complex coordination of multiple signaling pathways. This developmental transition is associated with dramatic physiological and structural changes, including hormone regulation, cell division, cell proliferation, and tissue differentiation. Auxin is well known for its ability to trigger fruit initiation and subsequent fruit growth independently from pollination. Exogenous IAA treatment of tomato ovary (cv. MicroTom) induces fruit set and early growth in a manner similar to that triggered by flower pollination. To investigate the extent to which the two types of fruit setting involve the same gene regulatory networks, we implemented a genome-wide transcriptomic profiling of the flower-to-fruit transition through deep sequencing. To prevent accidental self-pollination, tomato flowers were emasculated 1 day before anthesis and were either manually pollinated or treated with IAA, then sampled simultaneously at 4 DPA or 4 IAA (Hu et al., 2021).

Deep sequencing generated reads ranging from 23 to 33 million, depending on the sample, with 87%~89% of the reads being uniquely mapped to the *S. lycopersicum* genome (ITAG4.1). Gene expression values are provided as mean normalized counts per kilobase of transcript. Overall, the transcripts detected in 0 DPA, 4DPA, or 4 IAA tissues correspond to a total of 25,037 genes, representing 72% of the 34,688 tomato genes. Among these, 21,516 (62.0%) were expressed in all samples (**Supplementary Table S1**). Out of the 22,762 (65.6% of total tomato genes) expressed in 4 IAA samples, only 310 genes are specifically expressed in this tissue, while the expression of 905 genes (3.8%) is specific to 0 DPA samples, and 727 (3.1%) to 4 DPA fruits. Together, 1,548 genes were specifically expressed in young fruits (either in 4 DPA or 4 IAA fruits).

**Figure 1:**
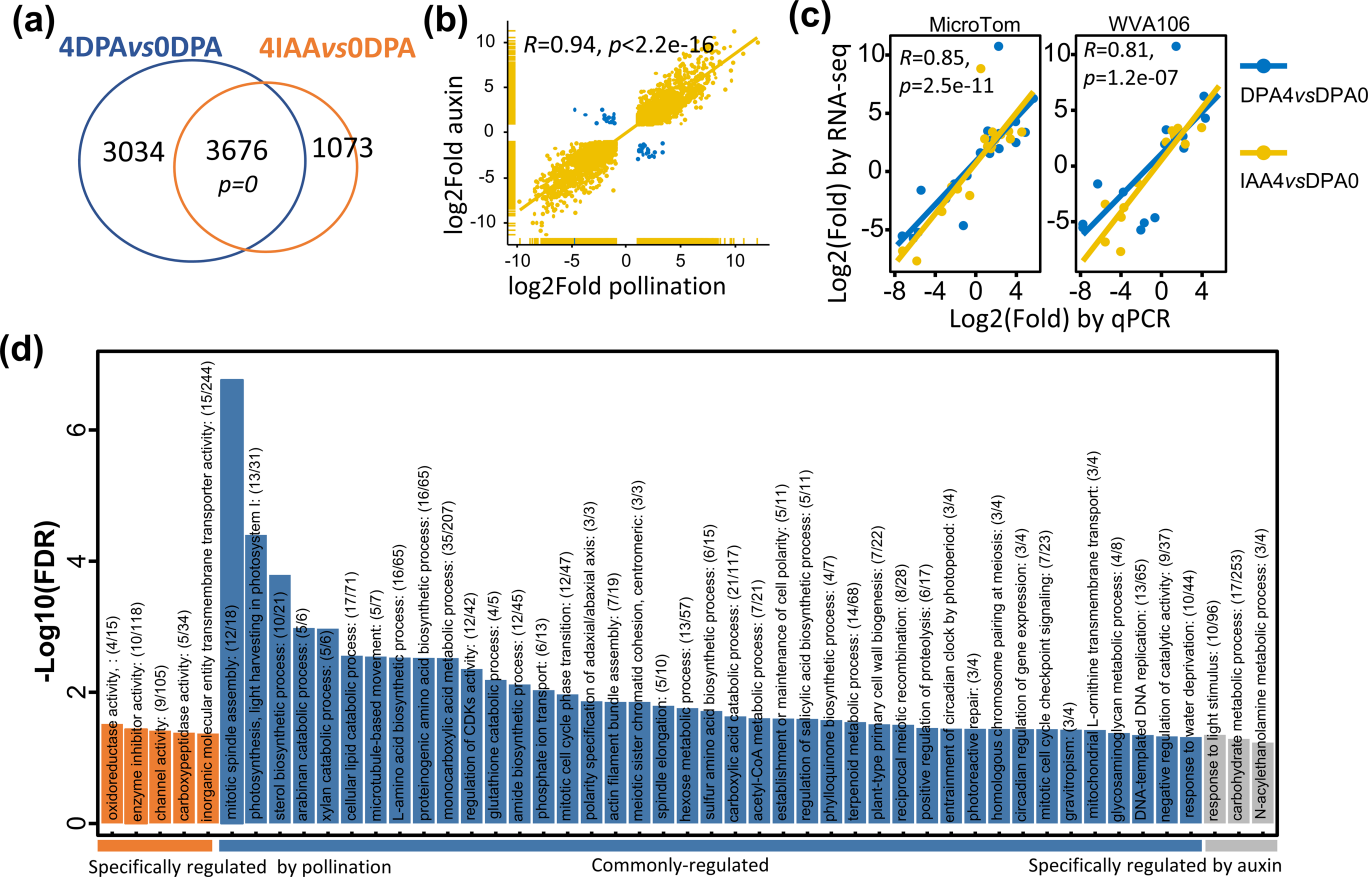
Genome-wide transcriptomic profiling of tomato genes during the fruit-set process induced by natural pollination and auxin induction. **(A)** Comparison of the number of differentially expressed genes during pollination- and auxin-induced fruit sets. Fold change > 2 and *padj* < 0.01. **(B)** Correlation of expression changes in common DEGs between pollination-induced and auxin-treated fruit sets. Statistical significance between the two gene datasets was evaluated using a hypergeometric test, with all expressed genes considered total size. **(C)** Correlation levels between RNA-seq and qRT-PCR expression data were assessed in two distinct tomato cultivars (MicroTom and Wva106). The statistical significance of the correlation between RNA-seq expression and qRT-PCR data was determined by a two-sided Pearson’s correlation test. **(D)** GO enrichment analysis for DEGs regulated by pollination or auxin during fruit set. Selected significantly enriched biological processes (BH-adjusted overrepresented *p*-value < 0.05) were annotated in the figure.

DEGs were identified using DESeq2 for raw count normalization. Considering a fold change ≥ 2 and an adjusted *p*-value < 0.01, a total of 6,710 and 4,749 genes were assigned as DEGs upon pollination and auxin treatment, respectively. Notably, a high proportion of DEGs (4,271 genes) were shared between pollination- and auxin-induced fruit (**Figure 1A**), and most of these DEGs showed the same trend of expression changes during the switch from flower to fruit (*R* = 0.94, **Figure 1B**), suggesting a largely similar transcriptomic reprogramming of the fruit set triggered by both auxin and pollination signals. In addition, qRT-PCR performed to validate the DEGs in both pollination- and auxin-induced fruit sets indicated that, among 16 randomly selected from DEGs, all exhibited a similar trend of expression changes for both pollination- and auxin-induced fruit sets (**Supplementary Table S2**; **Figure 1C**). Notably, this was further validated in a nondwarf cherry tomato cultivar, WVA106, which showed that 81% (13 genes) of the DEGs were consistently regulated during the two types of fruit set. These data indicated that the DEGs identified in our study are reliable for further analysis. GO analysis of the DEGs indicated that 41 GO terms were significantly enriched in both 4 DPA and 4 IAA samples. Among these, one-third of the biological processes are related to cell division and differentiation processes. Out of 44 DEGs related to cell division identified in the tomato genome, 55% (27 genes) are commonly induced by pollination and auxin, including cell cycle genes, cell division protein kinases (CDKs), and other regulators controlling cell division, consistent with the active cell division occurring at early stages of fruit development. Genes related to photosynthesis and carbohydrate metabolic processes were also differentially expressed in pollination- and auxin-induced fruits (**Figure 1D**), further supporting the idea that photosynthesis starts at a very early stage of fruit development in tomato. Importantly, a large number of common DEGs belong to the hormone signaling pathway, highlighting the critical role of hormones in regulating fruit set. Interestingly, several lipid-related processes, such as lipid oxidation, lipid catabolic processes, and cellular responses to lipids, were also significantly enriched. In summary, these data indicate that the common set of DEGs between auxin-induced and pollination-triggered fruit defines the fundamental processes required for fruit initiation in tomato.

Notably, a large proportion (45%, 3,034 genes) of DEGs are specific to pollination-dependent fruit sets, compared to only 22% that are specific to auxin-induced fruit set. GO analysis of pollination-specific DEGs further indicated that oxidoreductase, carboxypeptidase, and enzyme inhibitor activities were among the top-enriched biological processes, suggesting that more enzyme activity characterizes the pollination-triggered fruit set. On the other hand, GO terms enriched in 4 IAA DEGs are related to carbohydrate and *N*-acylethanolamine metabolic processes, suggesting that auxin treatment likely triggers more active sugar and lipid metabolism to support the fast growth of the fruit. Further examination of the DEGs at the tissue levels, based on previously reported LCM RNA-seq data (Pattison et al., 2015), showed a higher representation of embryo- and endosperm-preferentially expressed genes in pollination-induced fruit than in auxin-induced DEGs (**Table 1**). These data reflect the contrasted situation with regard to seed development in pollination-triggered fruit and auxin-treated fruit. Interestingly, individual investigation of these DEGs in embryo and endosperm tissues showed that their expression is promoted by pollination while repressed by auxin (**Supplementary Figures S1A, B**). By contrast, both auxin treatment and pollination lead to a relatively high proportion (from 21.30% to 30.43%, **Table 1**) of common DEGs observed in maternal tissues of the two types of young fruits, compared to embryo and endosperm tissues. These data indicate that in maternal tissues, the transcriptomic reprogramming relies on a set of genes largely common to pollination-induced and auxin-triggered fruits, whereas a highly contrasted situation prevails in embryo and endosperm tissues.

**Table 1:** Number of DEGs in different tissue-preferential gene sets.

Preferential tissues^a^	Gene Nb	Pollination-DEG		Auxin-DEG		Common-DEG^b^		Diff-DEG^c^	
		Nb	%	Nb	%	Nb	%	Nb	%
Embryo	1,535	204	13.29%	124	8.08%	93	6.06%	3	0.20%
Endosperm	591	121	20.47%	82	13.87%	53	8.97%	4	0.68%
Seed coat	457	224	49.02%	148	32.39%	125	27.35%	2	0.44%
Funiculus	385	132	34.29%	105	27.27%	82	21.30%	0	0.00%
Pericarp	731	261	35.70%	217	29.69%	166	22.71%	2	0.27%
Septum	966	426	44.10%	348	36.02%	294	30.43%	2	0.21%
Placenta	1,096	384	35.04%	295	26.92%	234	21.35%	1	0.09%

^a^Preferential tissues refer to tissue-preferentially expressed gene clusters adapted from previous tissue-specific transcriptomic data (Pattison et al., 2015): embryo, cluster 12; endosperm, cluster 19; seed coat, cluster 14; funiculus, cluster 21; pericarp, clusters 8 and 28; septum, clusters 27 and 29; and placenta, clusters 20 and 26.

^b^DEGs that exhibit common trends in changes induced by both pollination and auxin treatment.

^c^DEGs exhibit opposite changes between pollination and auxin treatments.

### Both pollination and auxin induce massive changes in the expression of hormone-related genes

**Figure 2:**
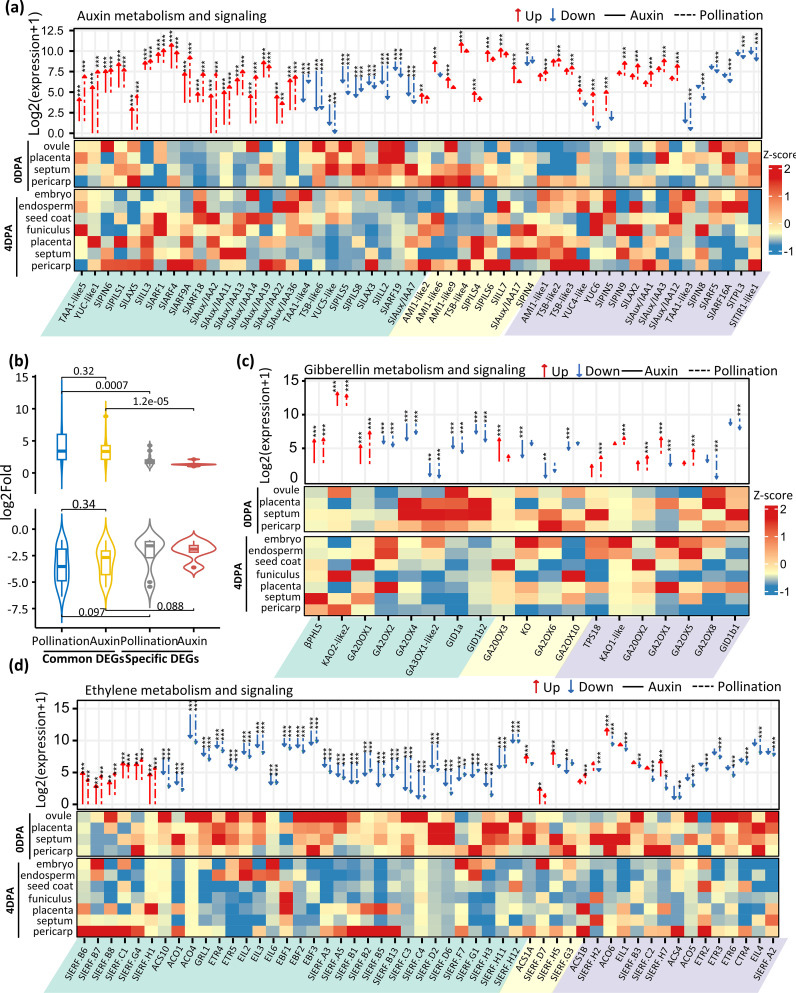
Differential expression of hormone-related genes during fruit set. DEGs associated with auxin **(A)**, gibberellin **(C)**, and ethylene **(D)** metabolism and signaling are shown. Gene expression is indicated as Log2 of mean normalized counts per kilobase +1. Common-, auxin-specific-, and pollination-specific DEGs are shaded in green, yellow, and purple, respectively. Dashed lines represent natural pollination, while solid lines indicate auxin treatment. Genes with significant differential expression were marked by asterisks. Gene expression (z-score) in selected tissue types from the ovary and pollination-induced young fruit is adapted from a previous study (Pattison et al., 2015) and displayed in the lower panel. **(B)** Average gene expression of common or type-specific DEGs associated with auxin metabolism and signaling. Genes with significant differential ex*p*ression were marked by asterisks ( ** Fold > 2 and 0.001< *p*-value < 0.01; *** Fold > 2 and *p*-value < 0.001).

Auxin and gibberellin are two critical hormones for fruit sets, but whether other hormones are also actively involved in this transition remains unclear. The large number of biological processes related to hormone regulation that are enriched in pollination- or auxin-induced fruit (**Figure 1D**) prompted us to investigate the expression changes of individual genes involved in hormone metabolism and signaling. We first generated the most comprehensive list of genes for each hormone category by performing a BLAST search with *Arabidopsis* orthologs (TAIR10) along with publicly available genes. Out of 120 auxin-related genes identified in the tomato genome, nearly half (52) were differentially expressed during either pollination- or auxin-induced fruit set. Genes involved in all aspects of auxin metabolism and responses (**Figure 2A**) were affected by these changes, including auxin synthesis (tryptophan aminotransferases and flavin monooxygenases), transport (SlPINs, SlLAXs, and SlPILSs), and signaling (Aux/IAAs and Auxin Response Factors). Among these DEGs, 50% were similarly regulated by both pollination and auxin induction, and their expression changes were significantly higher than those DEGs specific to auxin-treated or pollination-induced samples (**Figure 2B**). IAA is mainly synthesized from the amino acid tryptophan (Trp) in a two-step pathway by Tryptophan Aminotransferase of *Arabidopsis* (TAAs) and flavin monooxygenases (YUCCAs) or in an IAM-dependent pathway by indole-3-acetamide hydrolase (AMI) family. Consistently, *TAA1-like5* and *YUC-like1* were significantly upregulated in both pollination- and auxin-induced fruit, while *YUC4-like* and *YUC6* were only induced by pollination, in line with their specific expression in seed tissues. It is noteworthy that *YUC-like1* is highly and preferentially induced in the septum, suggesting that the septum is one of the main tissues contributing to overall auxin synthesis required for both pollination-dependent and pollination-independent fruit initiation in tomato. Although several TAAs, Trp synthases (TSBs), and YUCs were downregulated, these changes might be due to a sample dilution effect, considering that the upregulation of their expression occurs specifically in the embryo tissue of pollination-induced fruit. Alternatively, given that the embryo and endosperm tissues are absent in auxin-induced fruit, it might simply reflect a decreased expression of these genes in maternal tissues, since both pollination and auxin induction lead similarly to the downregulation of these genes (**Figure 2A**). On the other hand, a high number of ARFs and Aux/IAAs genes were activated during the fruit set process, including the previously reported ARF4 (Jones et al., 2002) and ARF9A (de Jong et al., 2015), which are involved in cell division. Consistently, the majority of these auxin signaling genes were expressed in maternal tissues, with *Aux/IAA2* being highly induced in the seed coat and septum, and *Aux/IAA 11* and *Aux/IAA 13* in the placenta. Altogether, these data indicate that high auxin activity is promoted in maternal tissues during both pollination-dependent and pollination-independent fruit set in tomato.

Nine out of the 19 GA-related DEGs were upregulated upon auxin or pollination, among which eight are involved in GA synthesis (**Figure 2C**), including two kaurene synthases (*TPS18* and beta-phellandrene synthase [*βPHLS*]), three kaurenoic acid oxidases (*KAO2-like2*, *KO* and *KAO1-like*), and three GA20 oxidases (*GA20ox1*, *GA20ox2*, *GA20ox3*). Among the 11 downregulated genes, six encode GA2 oxidases involved in reducing endogenous bioactive GA levels and three encode gibberellin receptors. Notably, all three GA20 oxidase genes were highly expressed in the funiculus of pollination-induced fruit, while most GA2OXs were depleted in the same tissue, consistent with highly active GA levels in the funiculus during fruit initiation. Altogether, the data support the idea that both pollination-dependent and pollination-independent fruit setting require active GA synthesis and signaling, and that exogenous auxin treatment promotes GA synthesis through a similar set of GA-related genes as the pollination-dependent fruit set.

Notably, out of 11 DEGs related to brassinosteroids, 10 showed significantly increased transcription in auxin-induced or natural pollination-induced fruit. Among these, eight brassinosteroid synthesis genes showed remarkably high expression, including *DET2*, *SMT1-like*, *SMT2-like3*, *delta14-sterol reductase*, *DWARF1-like*, *DWARF5-like2*, *HYD1-like*, and *STE1-like2* (**Supplementary Figure S2**). In particular, the steroid synthesis gene *DWARF1-like*, involved in the early C-22 hydroxylation pathway, displayed very high transcript levels in both 4 DPA and 4 IAA fruits. These data suggest that BR input also plays an important role in the control of fruit setting.

Strikingly, the downregulation of ethylene-related genes emerges as a major trend of the flower-to-fruit transition, with up to 41 genes (79%) of the 52 DEGs in this category showing significant downregulation (**Figure 2D**). Among these, 27 genes were downregulated by both pollination and auxin treatment, including six ethylene biosynthesis genes (1-Aminocyclopropane 1-Carboxylic Acid (ACC) *synthase* and three *ACC oxidases*), three ethylene perception genes (two *ETRs* and *GRL1*), three *EIN-like* genes, three *EBF* genes, and 15 ethylene response factor (*ERF*) genes. These data clearly emphasize the need for drastic repression of ethylene activity to allow the initiation of the fruit set process in tomato.

Genes involved in cytokinin synthesis are downregulated in both pollination and auxin-induced fruit. Among 20 DEGs in this category, 15 show a significant decrease in transcript levels, including adenosine phosphate-isopentenyl transferase genes (IPTs) (*IPT3-like* and *IPT5*), the rate-limiting enzymes for isopentenyladenine (iP) nucleotide synthesis. Consistently, cytokinin signaling and response genes also showed downregulation in pollination- or auxin-induced fruit, with 16 DEGs involved in cytokinin signal transduction and response. This indicates that cytokinin activity is also tuned down during the fruit initiation process (**Supplementary Figure S2**).

Moreover, genes related to abscisic acid, jasmonates, and salicylic acid undergo significant changes in their expression levels (**Supplementary Figure S2**). Several genes related to ABA (31 DEGs out of 85), JA (19 DEGs out of 45), and SA (17 DEGs out of 80) were also identified as differentially expressed. Overall, the data clearly support the idea that the fruit set process is under complex multihormonal control, with genes related to auxin, GAs, and BRs being mostly upregulated, whereas those related to ethylene are strongly downregulated, and those related to CK, JA, and ABA are rather tuned down.

### Auxin induces significant changes in histone marking similar to pollination-induced fruit set

**Figure 3:**
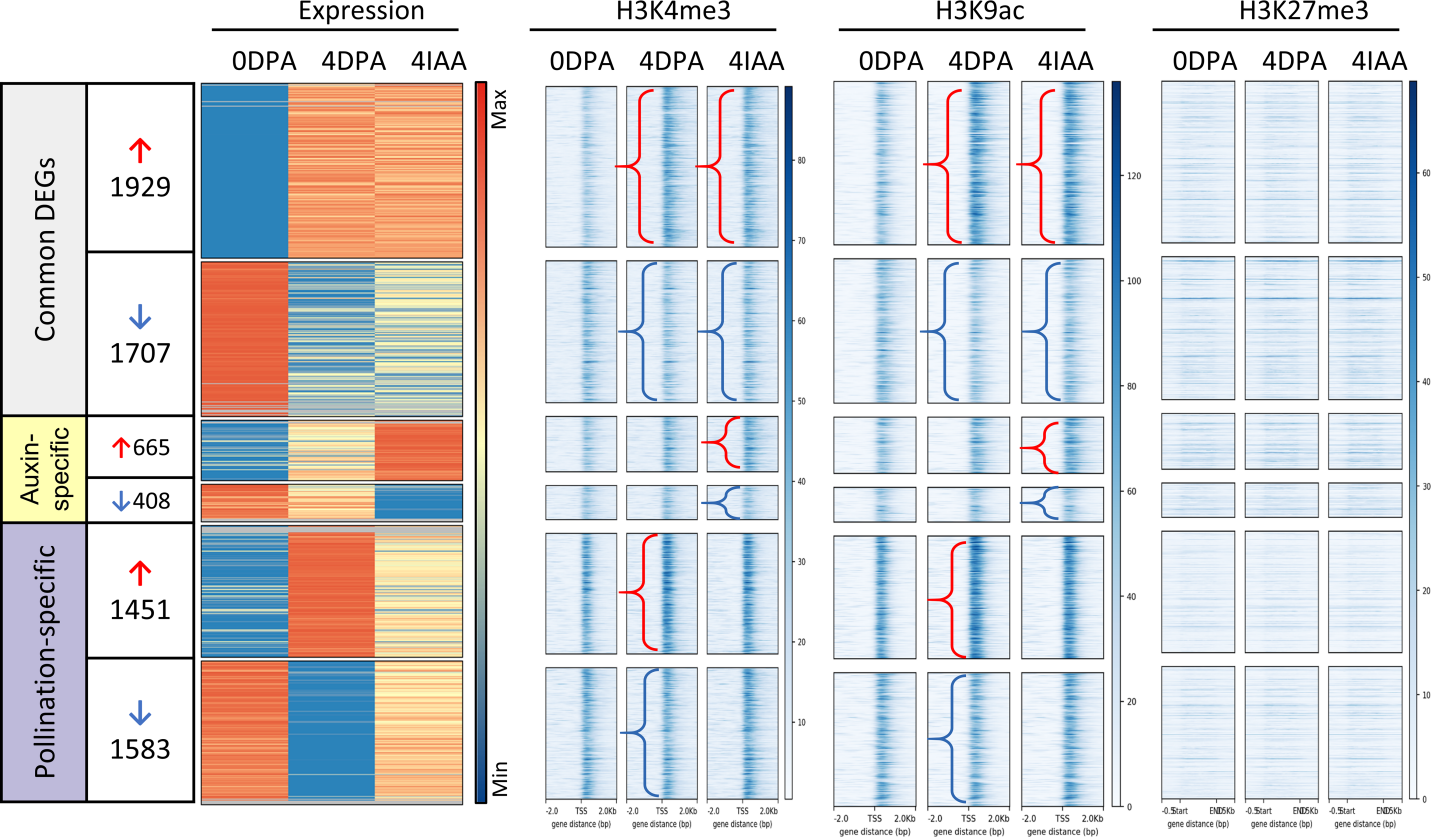
Profiles of gene expression and histone mark association for DEGs during fruit set. The density profile of gene expression (DE fold change > 2 and *p*-value < 0.01, first panel) is shown alongside changes in histone mark association from 0 to 4 DPA and 4 IAA tissues. Genes are ordered by the fold change of 4 DPA/0 DPA from high to low. Arrows indicate cases with differential histone mark association: red represents histone mark from 0 DPA to 4 DPA or 4 IAA, and blue represents the loss of histone marks.

We previously showed that a high proportion of DEGs in auxin-induced fruit set underwent similar changes in histone marking as those in pollination-dependent fruit set. To further explore the similarities and differences in histone marking between pollination-dependent and pollination-independent fruit sets, we separately profiled the association with three histone marks, including acetylation of lysine residue 9 (H3K9ac) and trimethylation of lysine residues 4 (H3K4me3) and 27 (H3K27me3), based on their changes in gene expression (**Figure 3**). The data showed a higher correlation between gene expression and association with the two permissive histone marks H3K4me3 and H3K9ac than the repressive mark H3K27me3. Notably, most of the common DEGs showed a clear correlation between changes in gene expression and changes in histone mark association.

Given that a large number of genes related to hormone regulation underwent changes in histone marks during pollination-induced fruit setting, we investigated whether auxin induces similar histone modifications. To address this issue, all DEGs related to the metabolism and signaling of auxin, gibberellin, ethylene, cytokinin, ABA, brassinosteroids, jasmonates, and salicylic acid were investigated for changes in their association with H3K9ac, H3K4me3, and H3K27me3.

**Figure 4:**
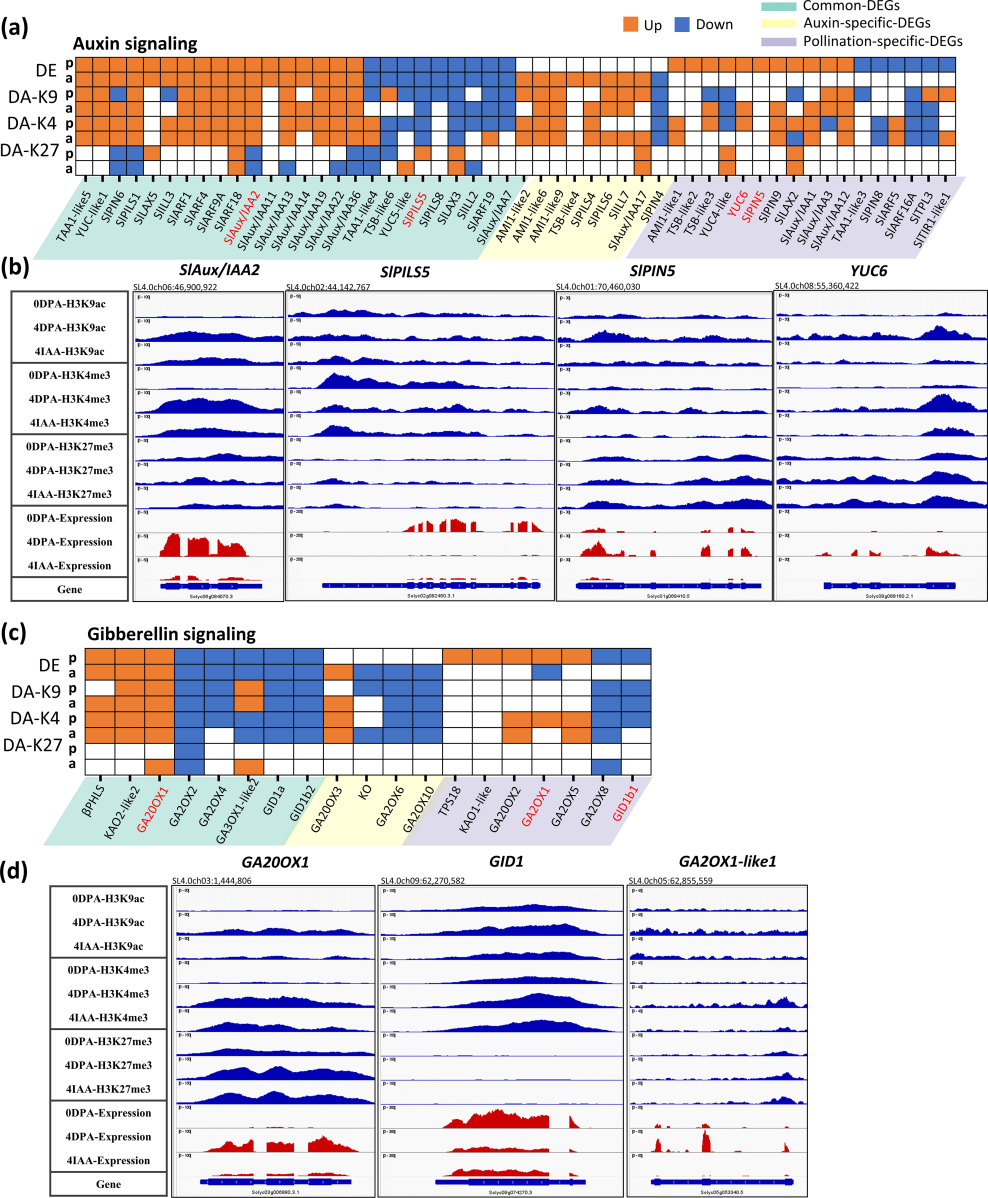
Differential expression and differential histone mark associations of auxin-related genes during fruit set. **(A, C)** Heatmap of DE and DAs (DA-K9, DA-K4, and DA-K27). Orange blocks indicate an increase in gene expression (fold > 2 and *p*-value < 0.01) or histone mark association (*p*-value < 0.01), while blue blocks indicate a decrease in gene expression or histone mark association. *p*, pollination-induced fruit set; *a*, auxin-induced fruit set. **(B, D)** Examples of differential mark association and gene expression of auxin- **(B)** and gibberellin-related **(D)** genes, visualized in IGV. Histone mark associations are marked in blue (top) and gene expression is marked in red (bottom).

Out of 52 DEGs associated with auxin metabolism and signaling identified in both types of fruit initiation, 48 showed differential association in at least one of the three histone marks (**Figure 4A**). Moreover, of the 16 DEGs common to pollination and auxin-induced fruit set, 26 displayed similar trends of changes in either H3K9ac or H3K4me3 histone marks. Notably, gaining either of these two histone marks correlated with increased gene expression, as exemplified by those involved in auxin synthesis, auxin transport, and auxin response. For instance, transcript accumulation of *SlAux/IAA2* was significantly increased in both auxin and pollination, along with their enrichment in H3K9ac and H3K4me3 marks and loss of H3K27me3 (**Figure 4B**). Along the same line, genes showing downregulation by both auxin- and pollination-induced fruit set display a clear decrease in active histone marks, as exemplified by *SlPILS5* (**Figure 4B**). Interestingly, it is remarkable that pollination dramatically enhanced (16 times) the expression of the *YUC6* gene (**Figure 4B**), with significant enrichment in H3K9ac and H3K4me3 marks. In contrast, auxin did not induce such a change in expression, suggesting that the induced expression of *YUC6* by natural pollination might occur specifically in the developing seeds, which are missing in auxin-treated fruits. *SlPIN5* also showed a distinct change in gene expression with pollination and auxin treatment (**Figure 4B**), indicating that these two inputs promote internal auxin transport in different ways. In keeping with the idea that H3K9ac and H3K4me3 histone marks are the major histone marks driving the changes in gene expression, these data indicate that pollination and auxin mostly trigger the same core set of auxin-related genes undergoing histone modifications during fruit set.

Most DEGs (17 out of 19) involved in gibberellin signaling, regulated by both pollination and auxin, displayed similar changes in at least one histone mark (**Figures 4C, D**). For example, pollination induced a gain in permissive H3K9ac and H3K4me3 marks on *GA20 oxidase 1* (*GA20OX1*; **Figure 4D**), resulting in significantly increased gene transcription. By contrast, auxin-induced enrichment in H3K4me3 but not in H3K9ac mark association. This indicates that pollination and auxin mark histone tails in slightly different ways, although H3K4me3 is the main driver modulating gene expression by both treatments. Moreover, compared to pollination, auxin specifically represses the association with H3K9ac and H3K4me3, accompanying the decreased transcription of *ent-kaurene oxidase* gene (**Figure 4C**), which encodes the enzyme catalyzing the early three-step oxidation required for gibberellin biosynthesis. This supports the idea that gibberellin is synthesized following the pollination-induced accumulation of auxin, which may promote GA biosynthesis in a later step, as evidenced by the significant increase in transcript levels of *KAO2-like2* and *GA20OX1* after pollination or auxin treatment.

Strikingly, almost all the DEGs (nine out of 11) related to brassinosteroid metabolism and signaling displayed a similar trend of increased H3K9ac and H3K4me3 histone marks in both pollination- and auxin-induced fruit initiation processes (**Supplementary Figure S3**). Notably, all these genes, with the exception of *BEH4-like2*, failed to show a change in H3K27me3-association during the fruit set, suggesting that their expressions are not hampered by the H3K27me3 repressive mark in the diverse tissues of young developing fruit. The data indicate that promoting brassinosteroid synthesis and signaling may make an important contribution to the control of the fruit set.

**Figure 5:**
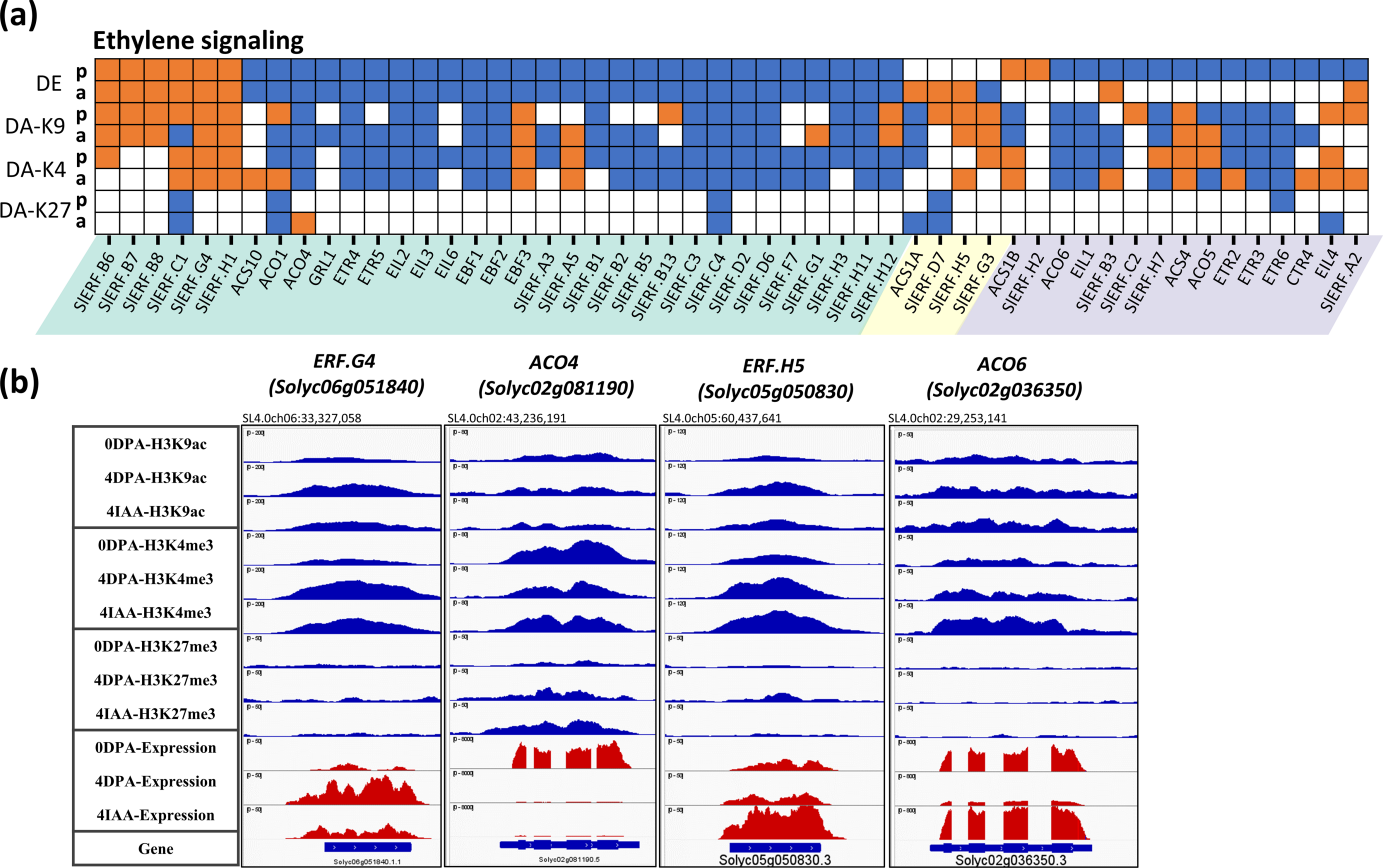
Differential expression and differential histone mark associations of ethylene-related genes during fruit set. **(A)** Heatmap of DE and DAs (DA-K9, DA-K4, and DA-K27). Orange blocks indicate an increase in gene expression (fold > 2 and *p*-value < 0.01) or histone mark association (*p*-value < 0.01), while blue blocks indicate a decrease in gene expression or histone mark association. *p*, pollination-induced fruit set; *a*, auxin-induced fruit set. **(B)** Examples of differential mark association and gene expression of ethylene-related genes, visualized in IGV. Histone mark associations are marked in blue (top), and gene expression is marked in red (bottom).

In accordance with the ethylene-related DEGs exhibiting mainly a downregulation trend during both auxin- and pollination-induced fruit set, their association with H3K9ac or H3K4me3 also mostly decreases during this developmental transition (**Figure 5A**). In total, out of 27 downregulated DEGs common to both pollination and auxin treatment, 18 show a similar trend of changes in either H3K9ac or H3K4me3 histone marks in the two types of fruit initiation processes. For example, *ACO4*, the main ACC oxidase gene expressed in ovules, displayed a net decrease in gene expression from 0 DPA to 4 DPA or 4 IAA stages, along with a dramatic loss of H3K9ac and H3K4me3 histone marks during this process (**Figure 5B**). The data reveal that both auxin and pollination similarly promote the flower-to-fruit transition by extensively lowering ethylene synthesis and perception. It is striking that all of the commonly upregulated DEGs are members of the *ERF* gene family, including *SlERF.B6-B8*, *SlERF.C1*, *SlERF.G4*, and *SlERF.H1*, which all show a significant increase in gene expression and H3K9ac or (and) H3K4me3 association after ovary fertilization and auxin treatment. This indicates that specific *SlERFs* might be activated during fruit set, but since ethylene biosynthesis genes are strongly downregulated, it can be assumed that the upregulation of these ERFs is not under direct regulation of ethylene. It is important to mention that although the expression of some genes is specifically regulated by auxin treatment or by pollination, their expression generally showed low fold changes and relatively low changes in histone mark association, supporting the hypothesis that auxin and pollination control flower-to-fruit transition mostly through the same subset of genes. The large number of genes related to cytokine, ABA, SA, and JA that display significant changes in histone marking and in their expression levels during pollination- and auxin-induced fruit setting (**Supplementary Figure S3**) clearly indicate that successful fruit initiation relies on a complex multihormonal control of the subordinated gene expression network.

### A large panel of transcription factor genes undergo changes in expression and epigenetic marks during the flower-to-fruit transition

**Figure 6:**
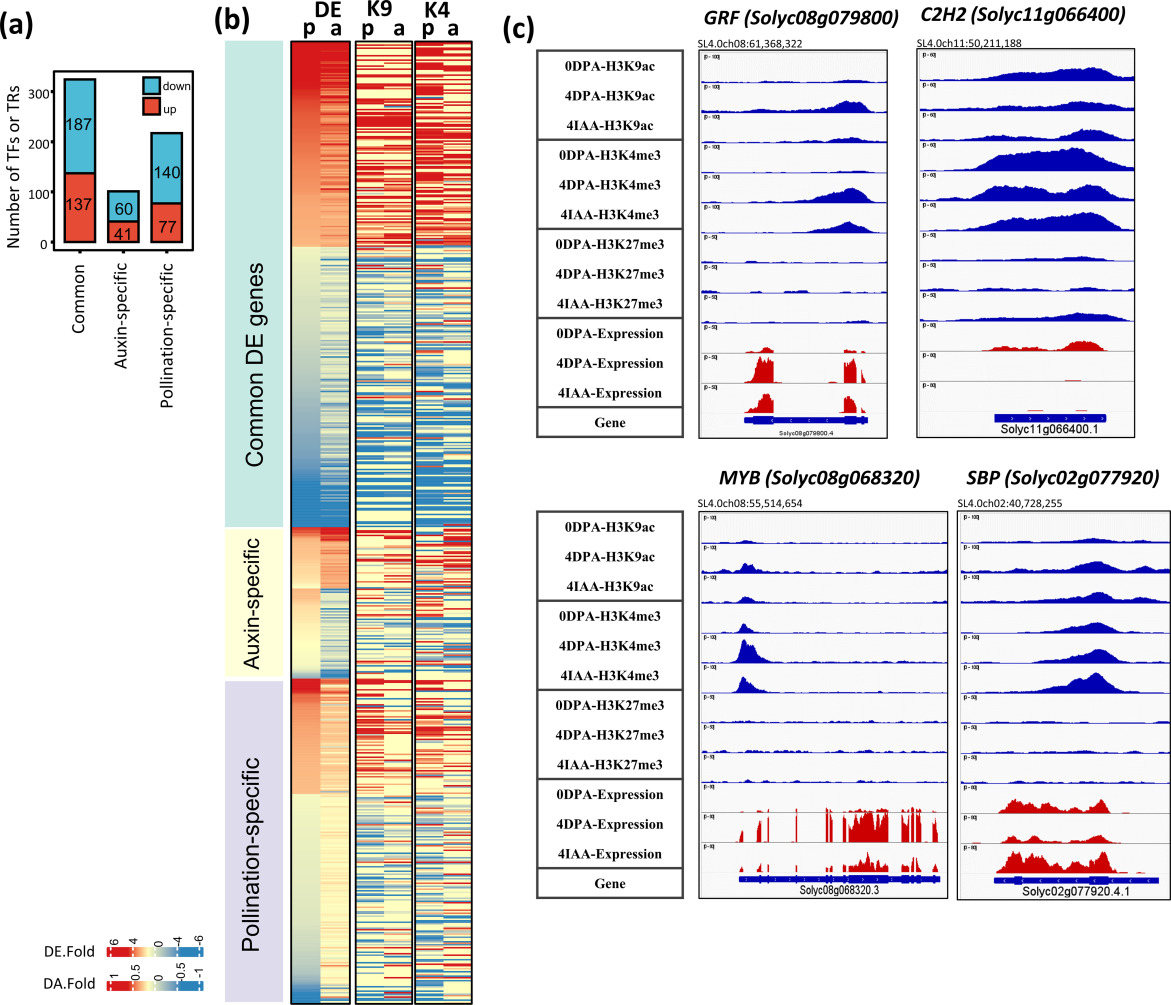
Differential expression and differential histone mark associations of TF genes regulated by both auxin and pollination. **(A)** Number of TF DEGs. **(B)** The heatmap of TF genes shows changes in gene expression and association with histone marks (DA-K9 and DA-K4). Red blocks indicate an increase in gene expression (fold > 2) or histone mark association by H3K9ac or H3K4me3 (*p*-value < 0.01); blue blocks indicate a decrease in gene expression or histone mark association. *p*, pollination-induced fruit set; *a*, auxin-induced fruit set. **(C)** Examples of differential mark association and gene expression of selected TFs, visualized in IGV. Histone mark associations are marked in blue (top), and gene expression is marked in red (bottom).

The large number (642 in total) of transcription factors displaying differential expression reflects the magnitude of transcriptomic reprogramming underlying the fruit-set process (**Figure 6A**). Remarkably, half (324) of these DEGs encoding TFs are commonly regulated by both pollination and auxin, while a smaller number are specifically regulated by auxin (41 upregulated and 60 downregulated) or pollination (with 77 upregulated and 140 downregulated).

To further understand the differences between auxin- and pollination-induced fruit setting we investigated the changes in gene expression and histone marking of individual TF genes in response to these two types of inputs. A similar proportion (93.9% by auxin induction and 92.8% by pollination induction) of TF DEGs showed differential association with at least one histone mark (**Figure 6B**). Notably, most of these TF genes were differentially associated with H3K9ac (71.1% of auxin-induced and 76.0% of pollination-induced) and H3K4me3 (77.8% and 80.4%) marks, while a lower proportion (32.2% and23.8%) displayed simultaneous changes in both active marks during fruit set. This is consistent with the critical role of active H3K9ac and H3K4me3 histone marks in driving gene transcription. Moreover, it is interesting to note that, in addition to the increased expression of *Aux/IAA* and *ARF* genes related to auxin activity, several other TF families displayed significant transcript elevation during fruit setting (**Supplementary Table S3**). These include *TCP* (six upregulated and two downregulated), *SBP* (four upregulated and two downregulated), *SNF2* (six upregulated and two downregulated), *GRF* (seven upregulated and one downregulated), *SET* (six upregulated), and *PhD* (three upregulated) family genes. Among these, several TFs showed a strong link with the fertilization and fruit development process. For example, two TCPs (*Solyc07g062680* and *Solyc03g116320*), whose homologs in *Arabidopsis* (TCP4 and TCP14) are required for endosperm development and activation of embryonic growth in seeds (Sarvepalli and Nath, 2011; Zhang et al., 2019), displayed increased transcript levels after pollination (by sevenfold) and auxin treatment (by four- to fivefold) (**Supplementary Table S3**). In addition, three *SBP*s (*Solyc01g090730*, *Solyc10g018780*, and *Solyc10g078700*) are significantly upregulated by both pollination and auxin treatment. Their strong expression levels in tomato carpels have been reported to play critical roles in early fruit development (Silva et al., 2014). Additionally, seven *GRF* TFs, involved in cell proliferation and cell expansion (Omidbakhshfard et al., 2015), were preferentially upregulated in 4 DPA or 4 IAA fruit. Among them, one (*Solyc08g079800*) showed a significant increase in H3K9ac (by pollination) and oH3K4me3 (by both pollination and auxin treatment) (**Figure 6B**).

The transcriptional increases of epigenetic regulation genes related to chromatin remodeling (SNF2 family) and histone methyltransferase (SET family) further emphasize the significance of the epigenetic modifications in this developmental transition. For instance, homologs of CHROMATIN REMODELING 1 (CHR1), which are involved in gene silencing and maintenance of DNA and histone methylation (*DDM1-like1* and *DDM1-like2*) (Osakabe et al., 2021), as well as *SDG30*, homologous to trithorax group proteins involved in H3K4me3 methylation in *Arabidopsis* (Alvarez-Venegas et al., 2003; Schuettengruber et al., 2011), displayed increase in their transcript levels during the fruit initiation process.

By contrast, genes belonging to several TF families exhibited a significant decrease in their transcript levels during both pollination-dependent and pollination-independent fruit initiation, such as those encoding C2H2 (10 upregulated and 24 downregulated), NAC (four upregulated and 20 downregulated), MADS-MIKC/M-type (18 downregulated), and PLATZ (four downregulated). These genes also displayed a significant loss of H3K9ac and/or H3K4me3 histone marks during fruit setting. For example, the C2C2 gene, *Solyc11g066400*, displayed a loss of histone marks during fruit setting (**Figure 6C**). Additionally, the expression of a NAC member (*Solyc07g045030*), whose ortholog in *Arabidopsis* encodes JUNGBRUNNEN1, a repressor of GA and BR biosynthesis (Shahnejat-Bushehri et al., 2017), was significantly reduced by both pollination (17 times) and auxin treatment (12 times) (**Supplementary Table S3**), further supporting the active role of GA and BR in fruit set. Furthermore, MIKC- and M-type MADS-box genes, including *SlDEF*, *AP3/PI*, and homologs of AGL62 (*Solyc01g10630*), AGL6 (*Solyc01g090960*), and AGL22 (*Solyc11g010570*) in *Arabidopsis*, also show significantly decreased transcript levels at 4 DPA and 4 IAA fruits. Notably, *SlAGL6*, one of the key regulators for parthenocarpic fruit formation in tomato (Klap et al., 2017), exhibited significant downregulation and a gain of H3K27me3 marks in both pollination-dependent and pollination-independent fruit setting. Given their expression pattern, these NAC and MADS-box TFs may function primarily as transcriptional repressors, acting as negative regulators of the onset of fruit formation.

The present study reveals that a majority (196 out of 217, 90.3%) of TF DEGs specifically induced by pollination were differentially associated with changes in at least one histone mark. Auxin treatment induced a similar proportion of TF DEGs, albeit a distinct subset displayed differential histone marking. Notably, while auxin modulates most TFs in the same way as pollination, the two types of fruit set can also diverge in their regulation of gene expression and histone marking. For instance, CNR, a critical regulator of fruit ripening that is repressed during the fruit development stages by DNA methylation, is specifically promoted by auxin, exhibiting a 2.5-fold increase in transcript levels and a gain in H3K4me3 mark. These data reveal that auxin uses a different pathway than natural pollination to promote gene transcription during fruit setting.

Compared to the 23.8% of TF DEGs differentially associated with H3K27me3 in pollination-induced fruit set, a higher proportion (32.2%) of TF genes underwent differential association with H3K27me3 in auxin-induced fruit formation (**Supplementary Table S3**). Of the TFs specifically induced by auxin, 38.6% were differentially marked by H3K27me3, whereas a lower proportion (19.8%) were found in pollination-specific regulated DEGs (**Supplementary Table S3**).

**Figure 7:**
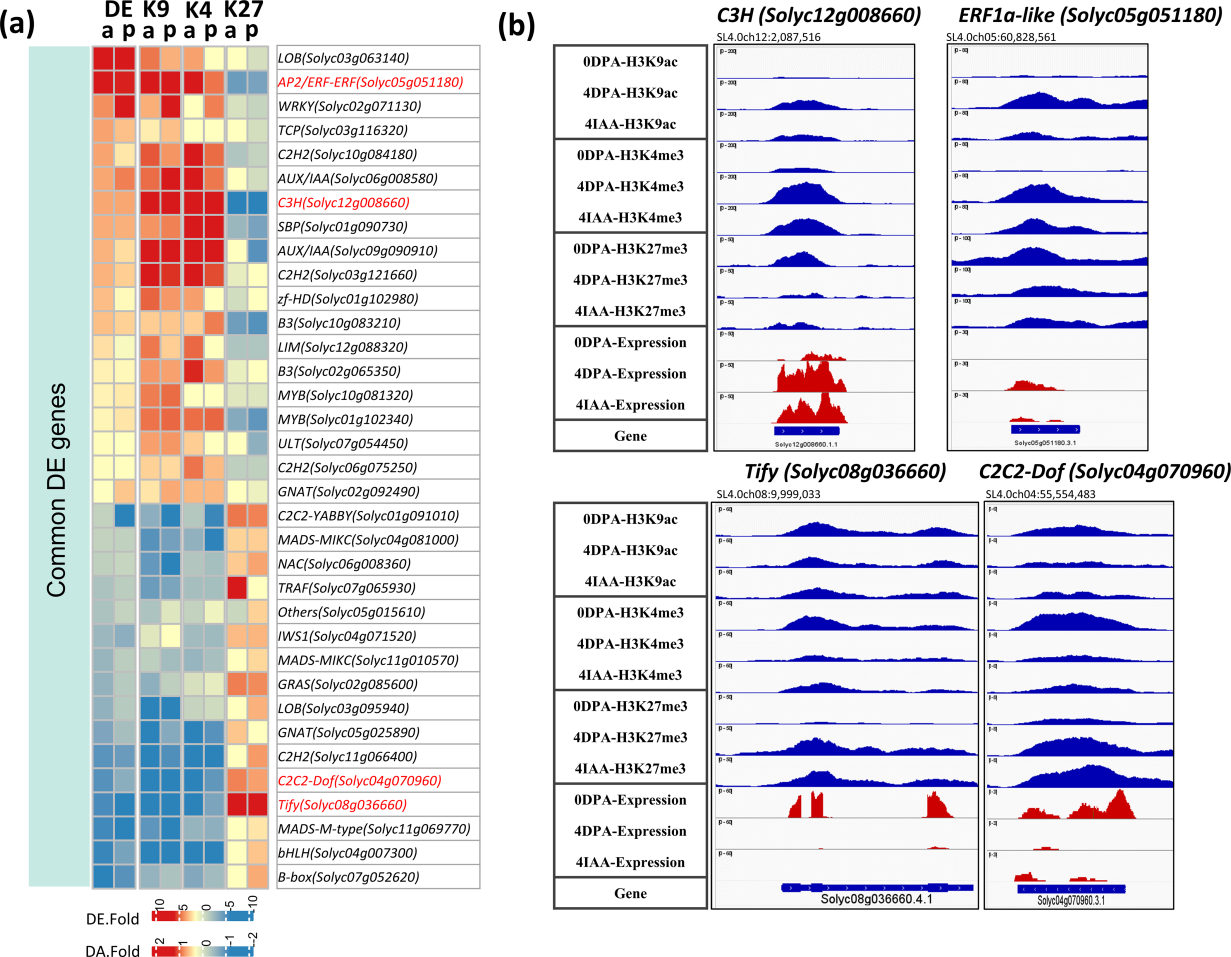
Differential expression and differential histone mark associations of TF genes regulated by both auxin and pollination. **(A)** Heatmap of commonly regulated DE TFs and their association changes with histone marks (DA-K9, DA-K4, and DA-K27). The red blocks indicate an increase in gene expression (fold > 2 and *p*-value < 0.01) or histone mark association by H3K9ac or H3K4me3 (*p*-value < 0.01); the blue blocks indicate a decrease in gene expression or histone mark association. *p*, pollination-induced fruit set; *a*, auxin-induced fruit set. **(B)** Examples of differential mark association and gene expression of selected TFs, visualized in IGV. Histone mark associations are marked in blue (top), and gene expression is marked in red (bottom).

TF DEGs displaying changes in all three histone marks are regarded as specific regulators of the fruit set. The data indicate that 35 TF DEGs showed consistent changes in all three histone marks (**Figure 7A**), with a gain of H3K9ac or H3K4me3 active marks and a loss of H3K27me3 repressive mark, or a loss of active marks associated with a gain of the repressive mark. This is exemplified by the changes in transcript levels of *C3H* (*Solyc12g008660*), *ERF1a-like* (*Solyc05g051180*), *Tify* (*Solyc08g036660*), and *C2C2-Dof* (*Solyc04g070960*), in which the three histone marks undergo significant modifications (**Figure 7B**).

## Discussion

**Figure 8:**
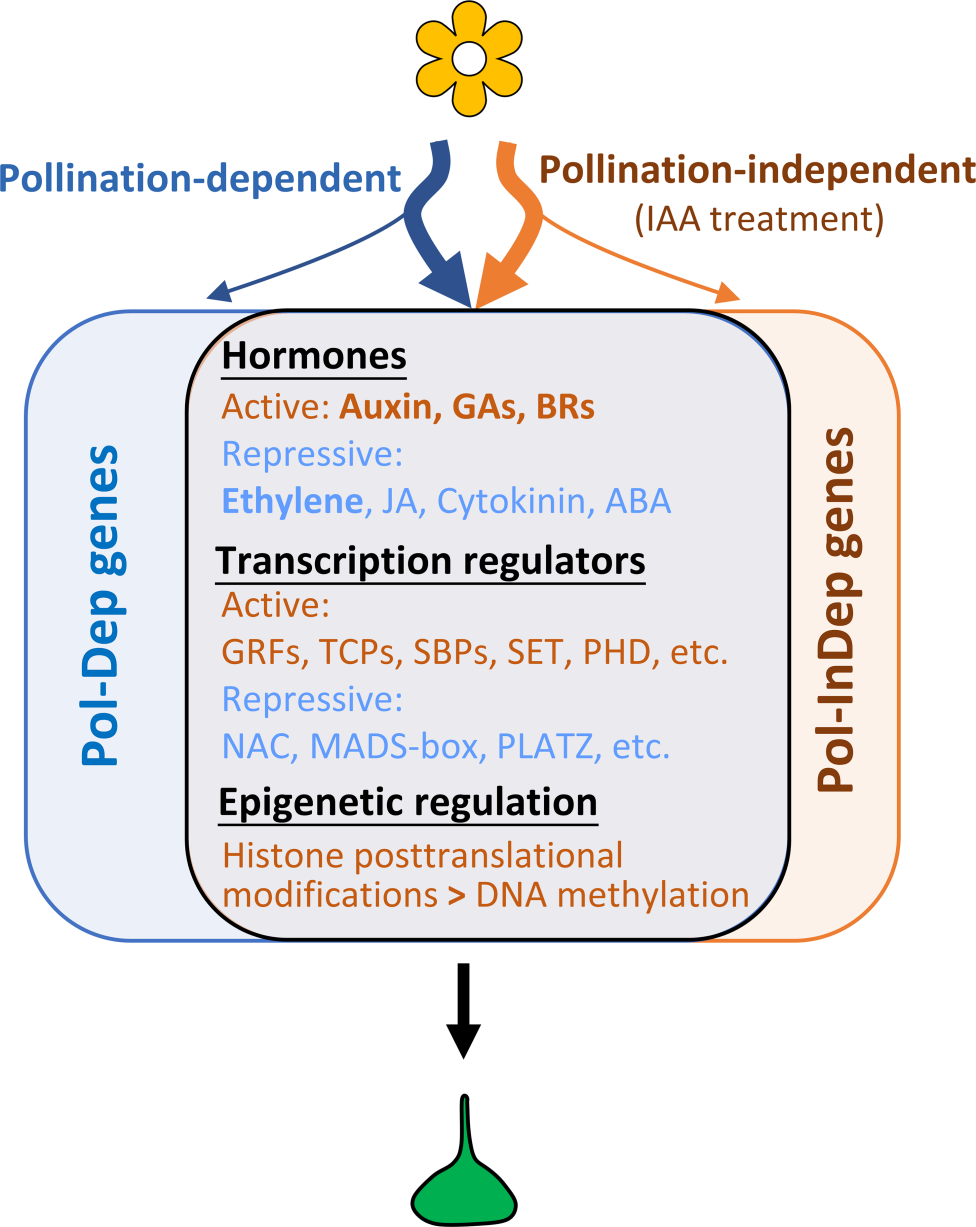
A schematic diagram of gene regulations underlying pollination-dependent and pollination-independent fruit set in tomato. A large number of genes related to hormones and transcription regulators exhibited similar trends in gene expression and histone marking in both pollination- and auxin-induced fruit sets, indicating largely similar genetic and epigenetic reprogramming of fruit set triggered by both auxin and pollination signals. Nevertheless, a significant number of genes are specifically triggered or repressed by auxin or pollination, suggesting that distinct pathways might also be involved in initiating the switch from flower to fruit in tomato. Among these, the genes related to seed development are specific to pollination-dependent fruit set, as this process is absent in auxin-induced fruit set.

Fruit set is a genetically programmed process, governed by the interaction between multiple hormonal signaling pathways and diverse transcriptional regulators, which coordinate a series of subordinate programs, including cell division, embryo development, photosynthesis, and epigenetic regulation. In the last decade, a large number of genes involved in fruit setting have been identified using diverse approaches, including cDNA-amplified fragment length polymorphism (AFLP), microarray, next-generation RNA sequencing, and studies in various mutants or hormone-treated fruits. However, the gene regulatory networks underlying this developmental shift, which is essential in determining crop yield, are still far from being fully understood. Our present study combining genome-wide transcriptomic profiling and ChIP-seq analysis, provides a comprehensive list of potential candidate genes associated with pollination-dependent and pollination-independent fruit sets. These findings may serve as novel resources for further deciphering the functional roles and contributions of these genes to fruit set. The data also revealed the role of auxin in triggering the transcriptomic reprogramming leading to the fruit set and showed that auxin operates in a manner largely similar to natural pollination in triggering the fruit initiation process in tomato (**Figure 8**). Common processes of cell division, including “DNA replication”, “cytokinesis”, “G2/M transition”, and “spindle assembly”, as well as of developmental processes, including “photosynthesis”, “flower development”, and “ovule development”, are enriched by both pollination and auxin treatment. Consistent with this, several transcription factor families involved in cell division or cell proliferation, such as *TCP* and *GRF* families, were significantly activated. Specifically, six out of eight *TCP* DEGs were significantly upregulated, in line with their conserved role in modulating cell cycle progression across various tissues and organs in diverse species (Huang and Irish, 2015; Resentini et al., 2015; Zhao et al., 2018, 2021; Ferrero et al., 2021; Gao et al., 2024). These findings suggest that TCPs contribute to fruit set and early fruit growth in both pollination- and auxin-induced fruit sets. It is noteworthy that in *Arabidopsis*, TCP homologs are also actively involved in hormone regulation. For instance, the *tcp14tcp15* double mutant has been shown to enhance gibberellin activity associated with cell division in the root apex (Resentini et al., 2015) and to regulate auxin activity in cell elongation (Ferrero et al., 2021). However, the precise functional roles of these TCPs in fruit set and fruit growth remain to be fully elucidated.

Ethylene and ABA have been proposed to play antagonistic roles to auxin and gibberellin during fruit set, keeping the ovary in a temporally protected and dormant state (Vriezen et al., 2007). Recent studies support the notion that ethylene suppresses tomato fruit set through the modification of gibberellin metabolism (Shinozaki et al., 2015). In addition, the knockout of EIN2, a key regulator of ethylene signaling, also leads to parthenocarpic fruit formation (Huang et al., 2022). In line with this view, the comprehensive exploration described in our present study indicates that the ethylene signaling pathway is significantly repressed in developmental ovaries and that the lower expression of genes involved in ethylene biosynthesis and activity is critical for early fruit initiation. This sharply contrasts with the increased ethylene levels in the abscission zone of flowers when fertilization fails. Altogether, these data open new possibilities for uncoupling hormone signaling pathways, beyond auxin and gibberellin, through cutting-edge genome editing strategies aimed at improving crop yield in fruit. For instance, manipulating the expression of ACC or ethylene biosynthesis genes prior to pollination could offer the potential to develop breeding lines with enhanced fruit set and reduced flower abscission.

Auxin and gibberellin are two central hormones for fruit initiation, and our data show that auxin synthesis (*YUCCAs*), transport (*PIN4*), signal transduction (*SlARF4*, *SlARF9*, and *SlARF18*), and response (AUX/IAA, GH3, and SAUR families) genes undergo change in their expression, associated with changes in H3K9ac or H3K4me3 histone marks. Consistently, *SlPIN4* and *SlARF9* have been reported to regulate fruit set in tomato, suggesting that modifications in histone marking on these auxin signaling genes are required for triggering the appropriate process of fruit development. Moreover, gibberellin synthesis genes were primarily upregulated and enriched in H3K9ac and H3K4me3 marks. This is clearly exemplified by the genes encoding *ent-*kaurenoic acid oxidase (*KAO2*) and GA 20-oxidase biosynthetic enzymes (*SlGA20ox1*, *SlGA20ox2*, and *SlGA20ox3*), which are significantly upregulated, while *SlGA2ox1* and *SlGA2ox2*, which encode GA-inactivating enzymes, were downregulated in ovaries at 4 days after pollination. This supports the idea that auxin-induced fruit set is partially mediated by gibberellin metabolism in tomato (Serrani et al., 2008). Similar to what is observed for the auxin signaling pathway, a large number of GA biosynthesis genes show changes in H3K27me3 marks. Unexpectedly, we did not detect a significant change in transcript levels of *SlIAA9*, *SlARF7*, *SlARF8*, and *DELLA*, all of which are known to be critical for fruit set (Wang et al., 2005, 2009; Goetz et al., 2007; Martí et al., 2007; de Jong et al., 2009b). This might be explained by the selected ovary stages and the use of whole ovary tissues for transcriptomic profiling. It has been shown previously that at the anthesis stage, *IAA9* expression localizes in the ovule, placenta, and funiculus, but is weak in the ovary wall and columella, gradually decreasing following pollination and spreading across the developing tissues (Wang et al., 2005). In summary, our data highlight the importance of chromatin modifications (H3K9ac and H3K4me3) and coordinated changes in transcript levels during the fruit set, whether induced by natural pollination or by auxin treatment. Nevertheless, it is anticipated that fast-developing spatial transcriptomic or single-cell resolution transcriptomic studies will provide, in the near future, a more precise picture of the genetic reprogramming underlying the transition from flower to fruit.

## Data Availability

The datasets presented in this study can be found in online repositories. The names of the repository/repositories and accession number(s) can be found in the article/**Supplementary Material**.
