# Supplementary figures and images for: Transcriptomic reprogramming and epigenetic regulation underlying pollination-dependent and auxin-induced fruit set in tomato
Source: Front Plant Sci. 2025 Feb 11;16:1495494. doi: 10.3389/fpls.2025.1495494 (PMC11850327; doi:10.3389/fpls.2025.1495494)

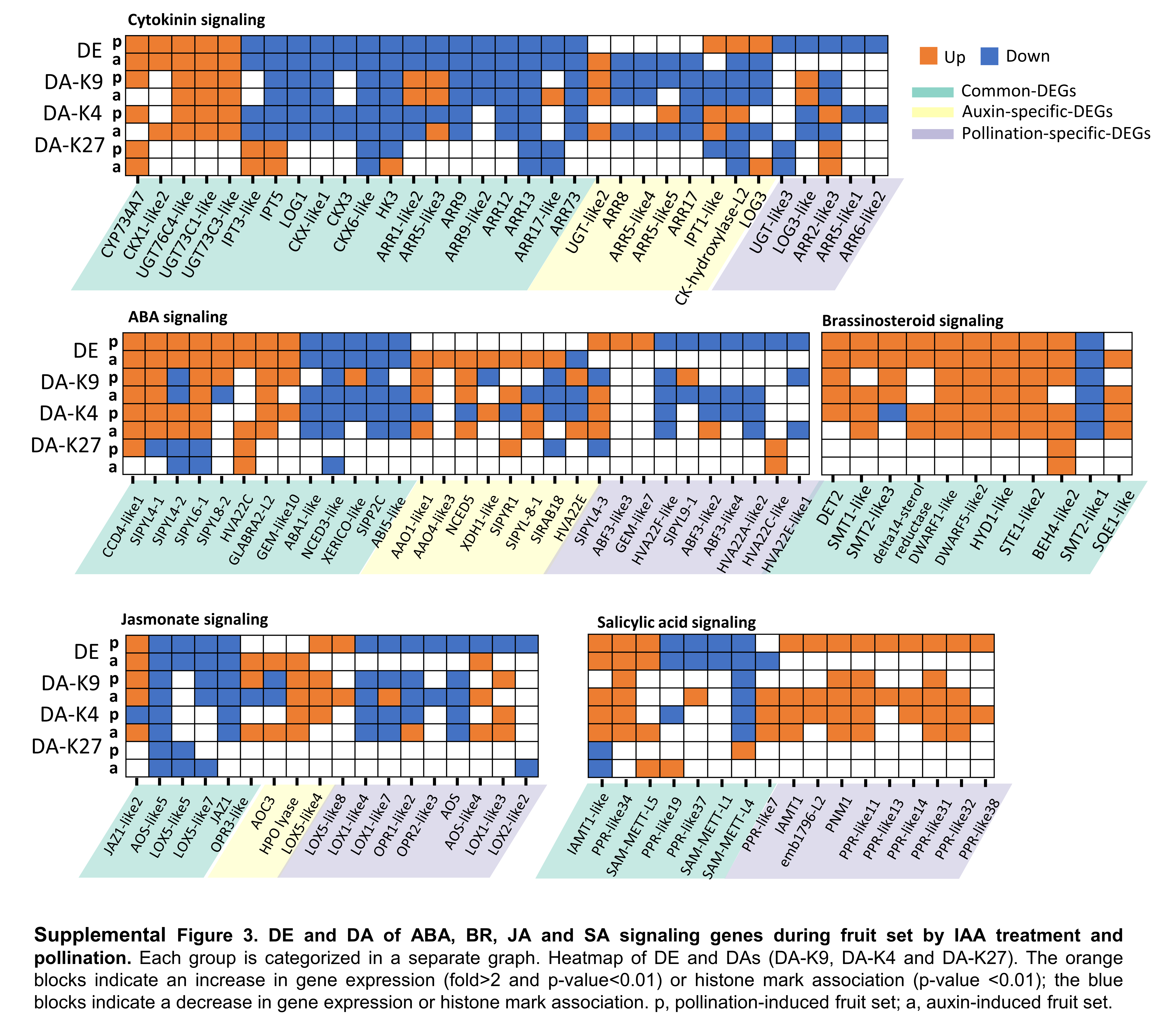

Supplement: Supplementary file 4 [file Image3.tif]
